# Supplementary material for: Natural plant products as potential inhibitors of RNA dependent RNA polymerase of Severe Acute Respiratory Syndrome Coronavirus-2
Source: PLoS One. 2021 May 13;16(5):e0251801. doi: 10.1371/journal.pone.0251801 (PMC8118514; doi:10.1371/journal.pone.0251801)
Supplement: S1 File — (DOCX) [file pone.0251801.s001.docx]

**Supplementary Data for “Natural plant products as potential inhibitors of RNA dependent RNA polymerase of Severe Acute Respiratory Syndrome Coronavirus-2”**

**S1 Fig. Methodology.** Protocol followed for creation of the phytochemical dataset with the list of the Indian medicinal plant sources.


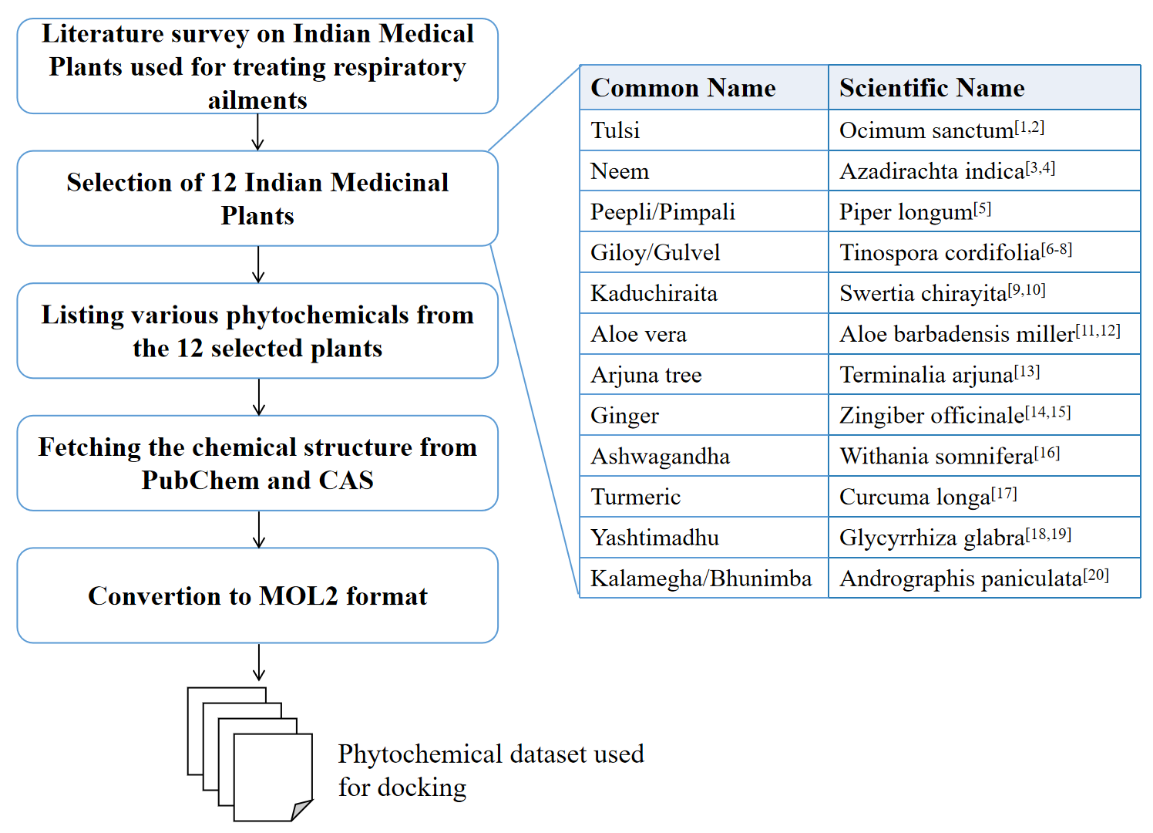


References numbered as superscripts beside the scientific name of the plants:

1. Saini A, Sharma S, Chhibber S. Induction of resistance to respiratory tract infection with Klebsiella pneumoniae in mice fed on a diet supplemented with tulsi (Ocimum sanctum) and clove (Syzgium aromaticum) oils. J Microbiol Immunol Infect. 2009 Apr;42(2):107-13. PMID: 19597641.
2. Cohen MM. Tulsi - Ocimum sanctum: A herb for all reasons. J Ayurveda Integr Med. 2014 Oct-Dec;5(4):251-9. doi: 10.4103/0975-9476.146554. PMID: 25624701; PMCID: PMC4296439.
3. Lee JW, Ryu HW, Park SY, Park HA, Kwon OK, Yuk HJ, Shrestha KK, Park M, Kim JH, Lee S, Oh SR, Ahn KS. Protective effects of neem (Azadirachta indica A. Juss.) leaf extract against cigarette smoke- and lipopolysaccharide-induced pulmonary inflammation. Int J Mol Med. 2017 Dec;40(6):1932-1940. doi: 10.3892/ijmm.2017.3178. Epub 2017 Oct 10. PMID: 29039495.
4. Jose Francisco Islas, Ezeiza Acosta, Zuca G-Buentello, Juan Luis Delgado-Gallegos, María Guadalupe Moreno-Treviño, Bruno Escalante, Jorge E. Moreno-Cuevas. An overview of Neem (Azadirachta indica) and its potential impact on health. Journal of Functional Foods, Volume 74, 2020, 104171, ISSN 1756-4646,doi: 10.1016/j.jff.2020.104171.\
5. Kumari M, Ashok BK, Ravishankar B, Pandya TN, Acharya R. Anti-inflammatory activity of two varieties of Pippali (Piper longum Linn.). Ayu. 2012 Apr;33(2):307-10. doi: 10.4103/0974-8520.105258. PMID: 23559810; PMCID: PMC3611634.
6. Saha S, Ghosh S. Tinospora cordifolia: One plant, many roles. Anc Sci Life. 2012 Apr;31(4):151-9. doi: 10.4103/0257-7941.107344. PMID: 23661861; PMCID: PMC3644751.
7. Sharma P, Dwivedee BP, Bisht D, Dash AK, Kumar D. The chemical constituents and diverse pharmacological importance of Tinospora cordifolia. Heliyon. 2019 Sep 12;5(9):e02437. doi: 10.1016/j.heliyon.2019.e02437. PMID: 31701036; PMCID: PMC6827274.
8. Antul, K., P. Amandeep, S. Gurwinder, and C. Anuj. “Review on Pharmacological Profile of Medicinal Vine: Tinospora Cordifolia”. Current Journal of Applied Science and Technology, Vol. 35, no. 5, June 2019, pp. 1-11, doi:10.9734/cjast/2019/v35i530196.
9. Kumar V, Van Staden J. A Review of Swertia chirayita (Gentianaceae) as a Traditional Medicinal Plant. Front Pharmacol. 2016 Jan 12;6:308. doi: 10.3389/fphar.2015.00308. PMID: 26793105; PMCID: PMC4709473.
10. Dey P, Singh J, Suluvoy JK, Dilip KJ, Nayak J. Utilization of Swertia chirayita Plant Extracts for Management of Diabetes and Associated Disorders: Present Status, Future Prospects and Limitations. Nat Prod Bioprospect. 2020 Dec;10(6):431-443. doi: 10.1007/s13659-020-00277-7. Epub 2020 Oct 28. PMID: 33118125; PMCID: PMC7648839.
11. Koul A, Bala S, Yasmeen, Arora N. Aloe vera affects changes induced in pulmonary tissue of mice caused by cigarette smoke inhalation. Environ Toxicol. 2015 Sep;30(9):999-1013. doi: 10.1002/tox.21973. Epub 2014 Feb 24. PMID: 24615921.
12. Zayas LE, Wisniewski AM, Cadzow RB, Tumiel-Berhalter LM. Knowledge and use of ethnomedical treatments for asthma among Puerto Ricans in an urban community. Ann Fam Med. 2011 Jan-Feb;9(1):50-6. doi: 10.1370/afm.1200. PMID: 21242561; PMCID: PMC3022046.
13. Shridhar Dwivedi, Terminalia arjuna Wight & Arn.—A useful drug for cardiovascular disorders, Journal of Ethnopharmacology, Volume 114, Issue 2, 2007, Pages 114-129, ISSN 0378-8741, <https://doi.org/10.1016/j.jep.2007.08.003.>
14. Townsend EA, Siviski ME, Zhang Y, Xu C, Hoonjan B, Emala CW. Effects of ginger and its constituents on airway smooth muscle relaxation and calcium regulation. Am J Respir Cell Mol Biol. 2013 Feb;48(2):157-63. doi: 10.1165/rcmb.2012-0231OC. Epub 2012 Oct 11. PMID: 23065130; PMCID: PMC3604064.
15. Anh NH, Kim SJ, Long NP, Min JE, Yoon YC, Lee EG, Kim M, Kim TJ, Yang YY, Son EY, Yoon SJ, Diem NC, Kim HM, Kwon SW. Ginger on Human Health: A Comprehensive Systematic Review of 109 Randomized Controlled Trials. Nutrients. 2020 Jan 6;12(1):157. doi: 10.3390/nu12010157. PMID: 31935866; PMCID: PMC7019938.
16. Choudhary B, Shetty A, Langade DG. Efficacy of Ashwagandha (Withania somnifera [L.] Dunal) in improving cardiorespiratory endurance in healthy athletic adults. Ayu. 2015 Jan-Mar;36(1):63-8. doi: 10.4103/0974-8520.169002. PMID: 26730141; PMCID: PMC4687242.
17. Rahmani AH, Alsahli MA, Aly SM, Khan MA, Aldebasi YH. Role of Curcumin in Disease Prevention and Treatment. Adv Biomed Res. 2018 Feb 28;7:38. doi: 10.4103/abr.abr_147_16. PMID: 29629341; PMCID: PMC5852989.
18. Samareh Fekri M, Poursalehi HR, Sharififar F, Mandegary A, Rostamzadeh F, Mahmoodi R. The effects of methanolic extract of Glycyrrhiza glabra on the prevention and treatment of bleomycin-induced pulmonary fibrosis in rat: experimental study. Drug Chem Toxicol. 2019 May 9:1-7. doi: 10.1080/01480545.2019.1606232. Epub ahead of print. PMID: 31072167.
19. Wang L, Yang R, Yuan B, Liu Y, Liu C. The antiviral and antimicrobial activities of licorice, a widely-used Chinese herb. Acta Pharm Sin B. 2015 Jul;5(4):310-5. doi: 10.1016/j.apsb.2015.05.005. Epub 2015 Jun 17. PMID: 26579460; PMCID: PMC4629407.
20. Okhuarobo A, Falodun JE, Erharuyi O, Imieje V, Falodun A, Langer P. Harnessing the medicinal properties of Andrographis paniculata for diseases and beyond: a review of its phytochemistry and pharmacology. Asian Pac J Trop Dis. 2014 Jun;4(3):213–22. doi: 10.1016/S2222-1808(14)60509-0. PMCID: PMC4032030.

**S2 Fig. Phytochemicals studies.** The 2D representations of the four phytochemicals, namely (A) swertiapuniside, (B) amarogentin, (C) sitoindoside IX and (D) cordifolide A.

**
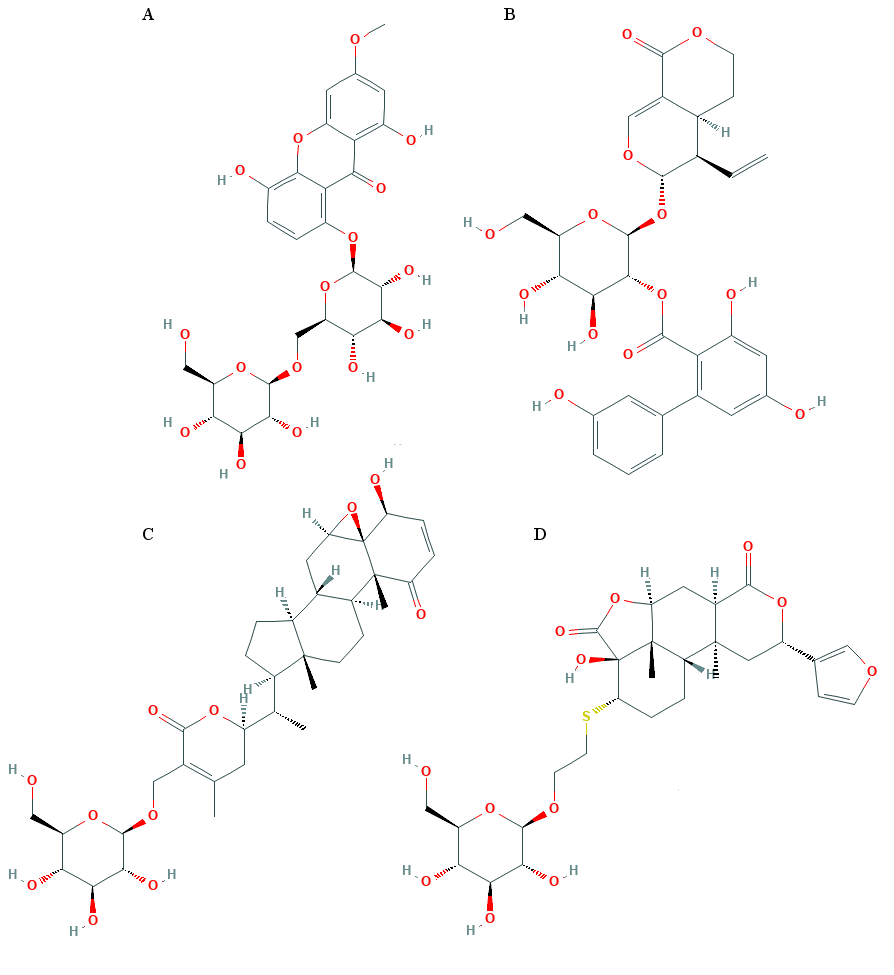
**

**S3 Fig. Residue fluctuations captured through PCA.** RMSF of the RdRP residues along the principal component 1(black), 2 (red), and 3 (green) for the systems RdRP-APO, RdRP-SWE, RdRP-AMR, RdRP-SIT and RdRP-COR.


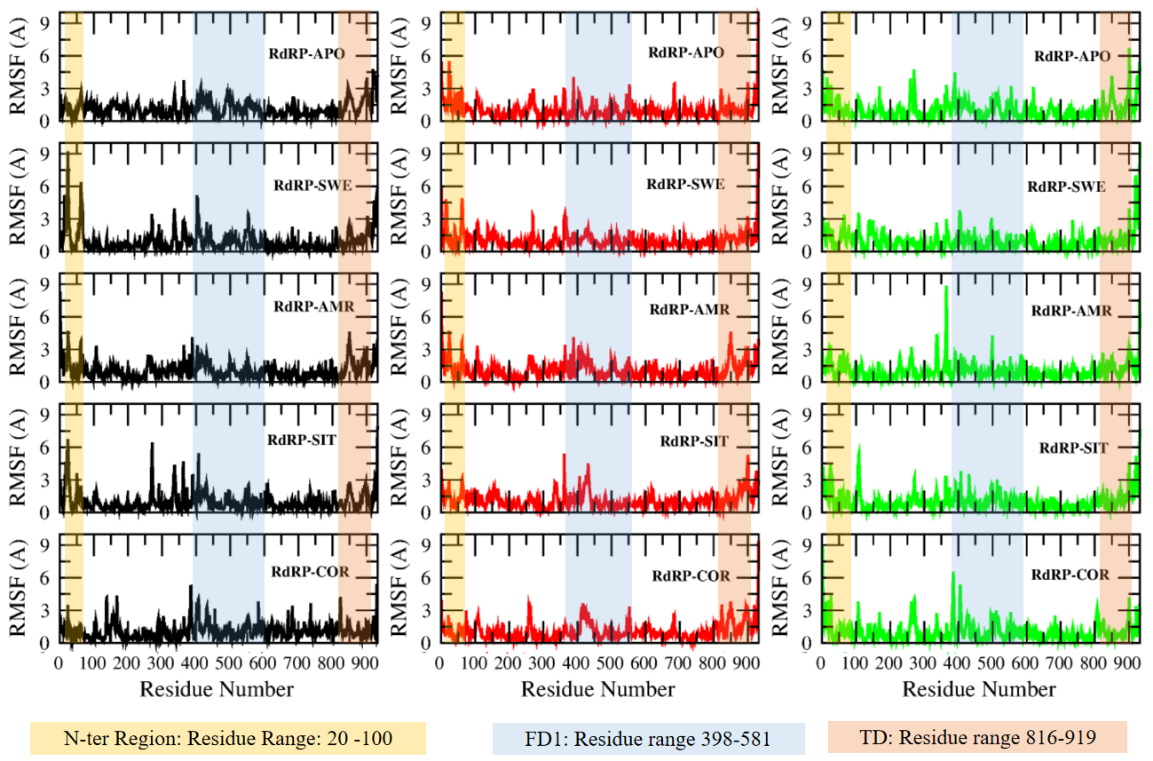


**S4 Fig. Free energy of binding.** Population distribution of the conformers obtained for the simulations based on the free energy of binding between the RdRP molecule and the corresponding phytochemical.


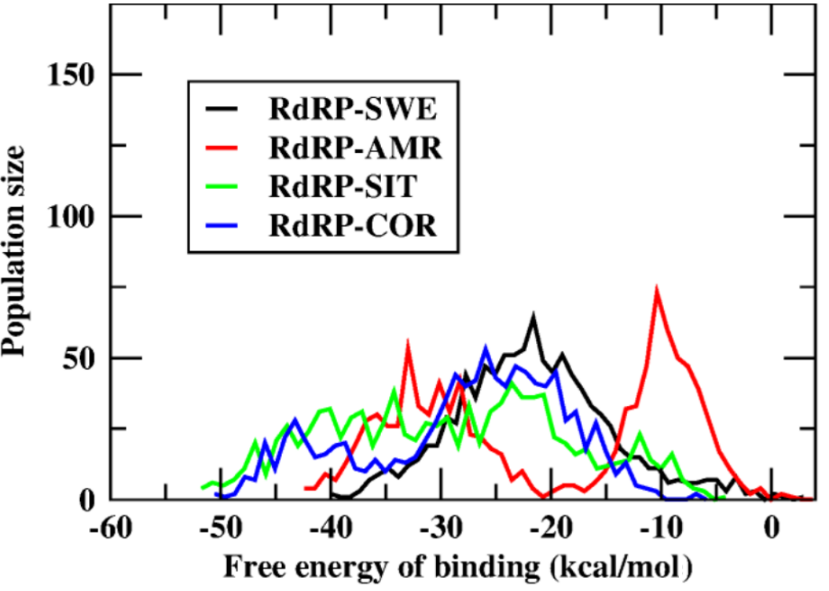


**S1 Table. ADMET properties.** ADMET properties of the four phytochemicals

|  |  | **ABSORPTION** | | | | **DISTRIBUTION** | | | **METABOLISM** | | | | | **TOXICITY** |
| --- | --- | --- | --- | --- | --- | --- | --- | --- | --- | --- | --- | --- | --- | --- |
| **Medicinal Plant (Source)** | **Phytochemical Name** | **GI** | **WS** | | **SP**  **(Log Kp)** | **BBB Crossing** | **Subcellular Localization** | **P-gly sub** | **hERG inb** | **CYP1A2 inb** | **CYP2C19 inb** | **A*CYP2D6 inb** | **CYP3A4 inb** | **CAR** |
| Swertia chirayita | Swertiapuniside | + | -1.64 | -11.04 | | - | Mitochondria | + | + | - | - | - | - | - |
|  | Amarogentin | + | -2.97 | -8.16 | | - | Mitochondria | - | - | - | - | - | - | - |
| Withania somnifera | Sitoindoside IX | + | -3.80 | -8.97 | | + | Mitochondria | + | + | - | - | - | - | - |
| Tinospora cordifolia | Cordifolide A | - | -3.542 | -9.7 | | - | Mitochondria | + | + | - | - | - | - | - |

The gastrointestinal absorption (GI), water solubility (WS) and skin permeation (SP) explains the absorption properties. Cordifolide A was observed to show low GI absorption, whereas, the remaining three showed favourable values for the same. The other two parameters showed allowed values for all the four phytochemicals. The blood-brain-barrier (BBB) crossing, subcellular localization and P-glycoprotein substrate (P-gly sub) parameters denote the distribution properties. All the phytochemicals were predicted to have the subcellular localization in the mitochondria. Except for amarogentin, all were predicted to serve as the P-glycoprotein substrate. Human ether-a-go-go Related Gene (hERG), cytochrome 1A2 (CYP1A2), cytochrome 2C19 (CYP2C19), cytochrome 2D6 (CYP2D6) and cytochrome 3A4 (CYP3A4) inhibitory property was calculated to understand the metabolism of the phytochemicals. Neither of them were predicted to be CYP inhibitors nor possessing toxic properties.

**S2 Table. Bond distance of hydrogen bonds.** The average bond distance for the hydrogen bond formed between RdRP residues and SWE, AMR, SIT and COR

| **RdRP Residue** | **Hydrogen Bond Length (Å)** |
| --- | --- |
| **Swertiapuniside (SWE)** | |
| ASP 760 (C) | 2.99 |
| ASP 761 (C) | 2.84 |
| SER 759 (C) | 2.85 |
| LYS 551 (F) | 3.5 |
| ASP 618 (A) | 3.28 |
| ARG 555 (F) | 3.4 |
| GLU 811 (E) | 2.81 |
| SER 814 (E) | 2.98 |
| ARG 553 (F) | 2.65 |
| LYS 798 (PD) | 2.88 |
| **Amarogentin (AMR)** | |
| LYS 621 (A) | 2.99 |
| ARG 553 (F) | 3.45 |
| ASP 452 (FD1) | 2.66 |
| ARG 555 (F) | 3.3 |
| ASP 618 (A) | 3.22 |
| ASP 760 (C) | 3.38 |
| **Sitoindoside IX (SIT)** | |
| ARG 555 (F) | 2.82 |
| ASP 684 (B) | 2.63 |
| SER 682 (B) | 2.67 |
| ALA 550 (F) | 2.95 |
| SER 549 (F) | 2.71 |
| GLY 683 (B) | 2.8 |
| ALA 558 (FD1) | 3.13 |
| **Cordifolide A (COR)** | |
| TRP 800 (PD) | 3.09 |
| LYS 798 (PD) | 2.42 |
| HIE 810 (PD) | 2.3 |
| ASP 618 (A) | 3.21 |
| SER 814 (E) | 2.84 |
| GLU 811 (E) | 2.99 |
| ARG 555 (F) | 3.4 |
| ASP 761 (C) | 2.79 |
| GLY 616 (A) | 2.82 |
| ARG 553 (F) | 3.17 |
